# Ischemic colitis and atrial septal aneurysm as a potential cause of systemic thromboembolism

**DOI:** 10.1002/ccr3.3889

**Published:** 2021-02-10

**Authors:** Eihab A. Subahi, Narinder Kumar, Abdulwahab M. Hamid, Mohamed S. Elmahadi, Mohamed A. Yassin

**Affiliations:** ^1^ Department of Internal Medicine Hamad Medical Corporation Doha Qatar; ^2^ Department of cardiology Alkhor Hospital Hamad Medical Corporation Doha Qatar; ^3^ Department of Gastroenterology and Hepatology Alkhor Hospital Hamad Medical Corporation Doha Qatar; ^4^ Department of General Medicine Alkhor Hospital Hamad Medical Corporation Doha Qatar; ^5^ Department of Medical Oncology National Center for Cancer Care and Research Hamad Medical Corporation Doha Qatar

**Keywords:** aneurysm, colitis, thromboembolism

## Abstract

Atrial septal aneurysm is a rare cardiac abnormality with thromboembolic potential and should be considered in a patient with ischemic colitis with no obvious risk factors.

## Graphical abstract

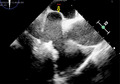

## 1 INTRODUCTION

Atrial septal aneurysm is a rare cardiac abnormality that is usually detected during routine echocardiography or during evaluation of thromboembolism cases. It may be isolated or associated with other cardiac defects, most often with patent foramen ovale. Atrial arrhythmias and arterial embolisms are associated complications that must be treated with anticoagulants. Herein, we report the case of a 62‐year‐old man who presented with abdominal pain and rectal bleeding and found to have ischemic colitis and atrial septal aneurysm on transesophageal echocardiography. The patient was treated with anticoagulation and rivaroxaban. Systemic thromboembolism is one of the complications of atrial septal aneurysm, and most of the reported cases are associated with stroke, transient ischemic attack, and renal emboli.

An atrial septal aneurysm is defined as redundant and mobile interatrial septal tissue in the region of the fossa ovalis with a phasic excursion of at least 10‐15 mm during the cardiorespiratory cycle.^1^ It can be classified according to its intrusion into the left or right atrium and motion during the respiratory cycle.^1^ The aneurysm may either protrude preeminently into the right or left atrium or produce striking oscillation into the atrial cavities during respiration according to variations in the pressure differences between the atria.^1^, ^2^ Atrial septal aneurysm is most commonly discovered incidentally during a routine evaluation, but in some populations, it can be associated with systemic thromboembolism ^1^, ^3^, ^4^ and intracardiac shunt; if it is associated with one or more atrial septal defects, the most common association is patent foramen ovale.^5^

Atrial septal aneurysm is usually diagnosed through transthoracic echocardiography; however, transesophageal echocardiography is more sensitive as the interatrial septum can be visualized more consistently.^6^

## 2 CASE PRESENTATION

Herein, we present the case of a 62‐year‐old man who presented to our facility with a one‐day history of severe lower abdominal pain associated with fresh bleeding per rectum. The patient had a long‐time history of hypertension and diabetes, which were controlled with medications. Upon presentation, the patient was in pain but conscious, alert, oriented, afebrile, and vitally stable. The abdominal examination findings were soft and lax with mild lower abdominal tenderness, and digital rectal examination was positive for blood. Cardiac examination results were normal. His blood tests showed mild neutrophilic leukocytosis and a normal hemoglobin level. Other laboratory results were within normal limits. Autoimmune and thrombophilia workups were both negative and viral serology tests for hepatitis B and C and human immunodeficiency virus were negative.

**FIGURE 1:**
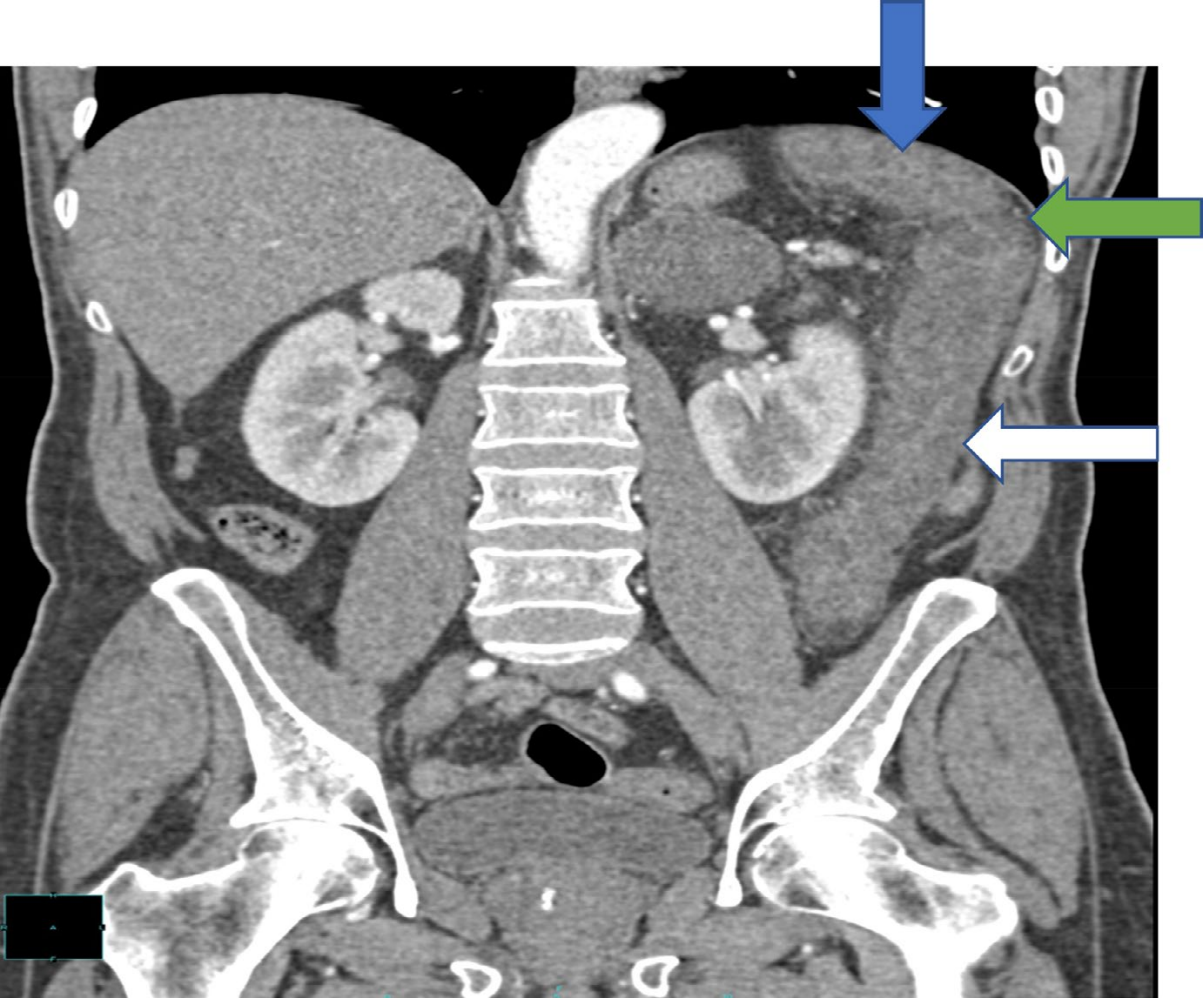
Diffuse circumferential wall thickening of the large colon involving the distal third of the transverse colon (blue arrow), splenic flexure (green arrow), and the entire descending colon (white arrow), associated with reduce mural enhancement and surrounding pericolonic fat stranding

**FIGURE 2:**
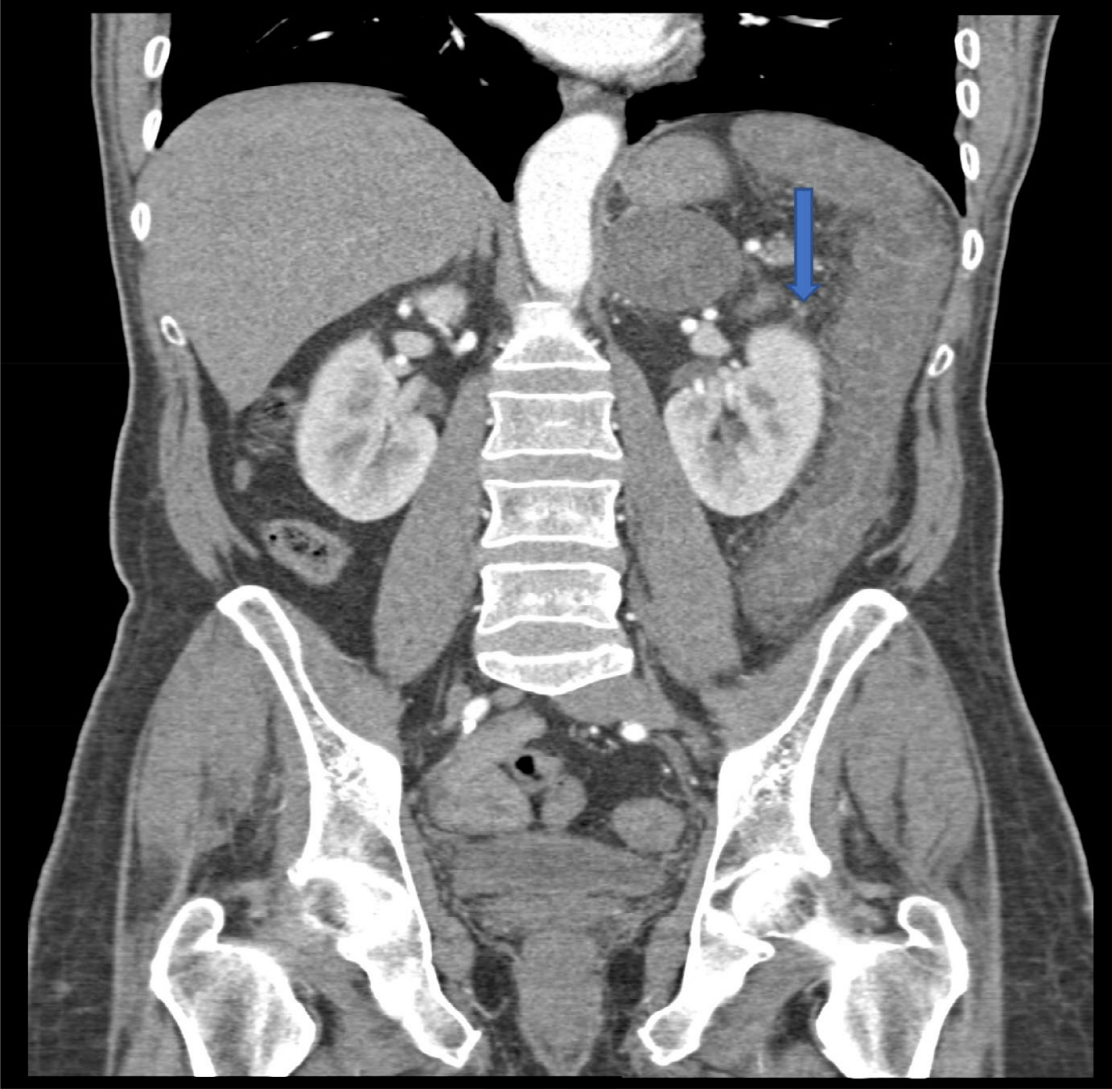
Patent main left colic artery, with poor contrast filling of its distal branches (blue arrow), poorly perfused vas recta, and associated overall poor bowel wall enhancement

**FIGURE 3:**
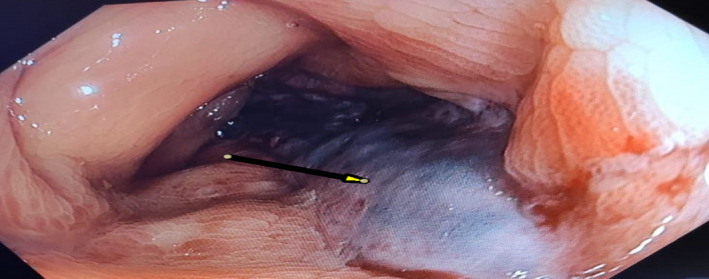
Yellow arrow shows the bluish colonic mucosa suggesting ischemic colitis

**FIGURE 4:**
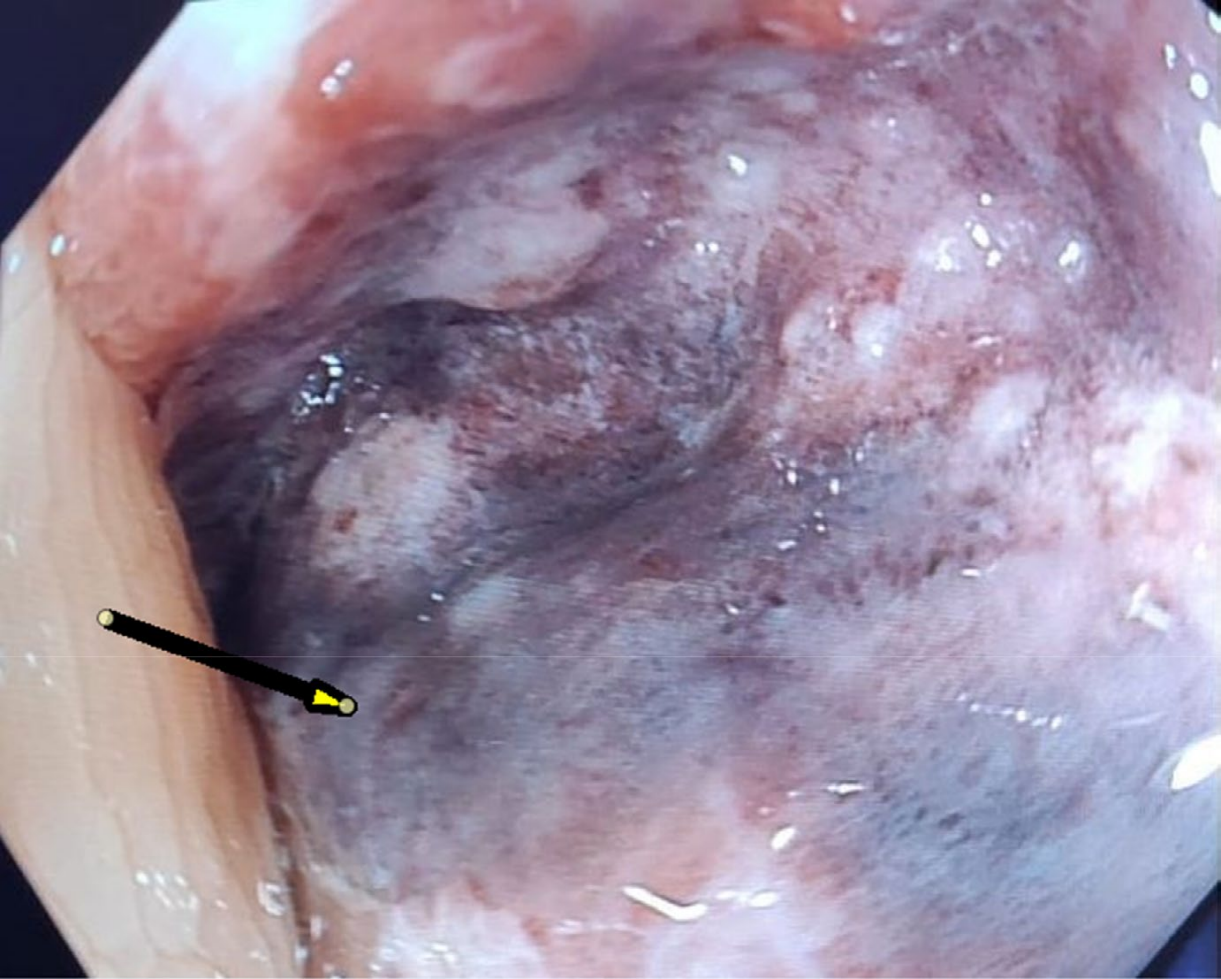
Yellow arrow marks the bluish colonic mucosa suggesting ischemic colitis

A contrast‐enhanced computed tomography (CT) of the abdomen and pelvis showed diffused colitis involving the entire descending colon with pericolonic fat stranding and possible reduced bowel wall perfusion with poor contrast filling of the distal branches of the left colic artery but without major vascular occlusions or stenosis, which was possibly accompanied by ischemic colitis (Figures 1 and 2). Colonoscopy showed a bluish mucosa with ischemia. Biopsy confirmed the diagnosis of ischemic colitis, which showed necrotic bowel mucosa with fibrin thrombi and fibrinous material (Figures 3 and 4). The patient underwent 12‐lead electrocardiography, which showed sinus rhythm and no arrhythmias. Transthoracic echocardiography detected mild mitral valve regurgitation, mild to moderate tricuspid valve regurgitation, and suspicion of an atrial septal aneurysm with no obvious shunt across it. Bubble contrast echocardiography did not reveal shunting across the atrial septum, and the left atrium was clear with no thrombus.

**FIGURE 5:**
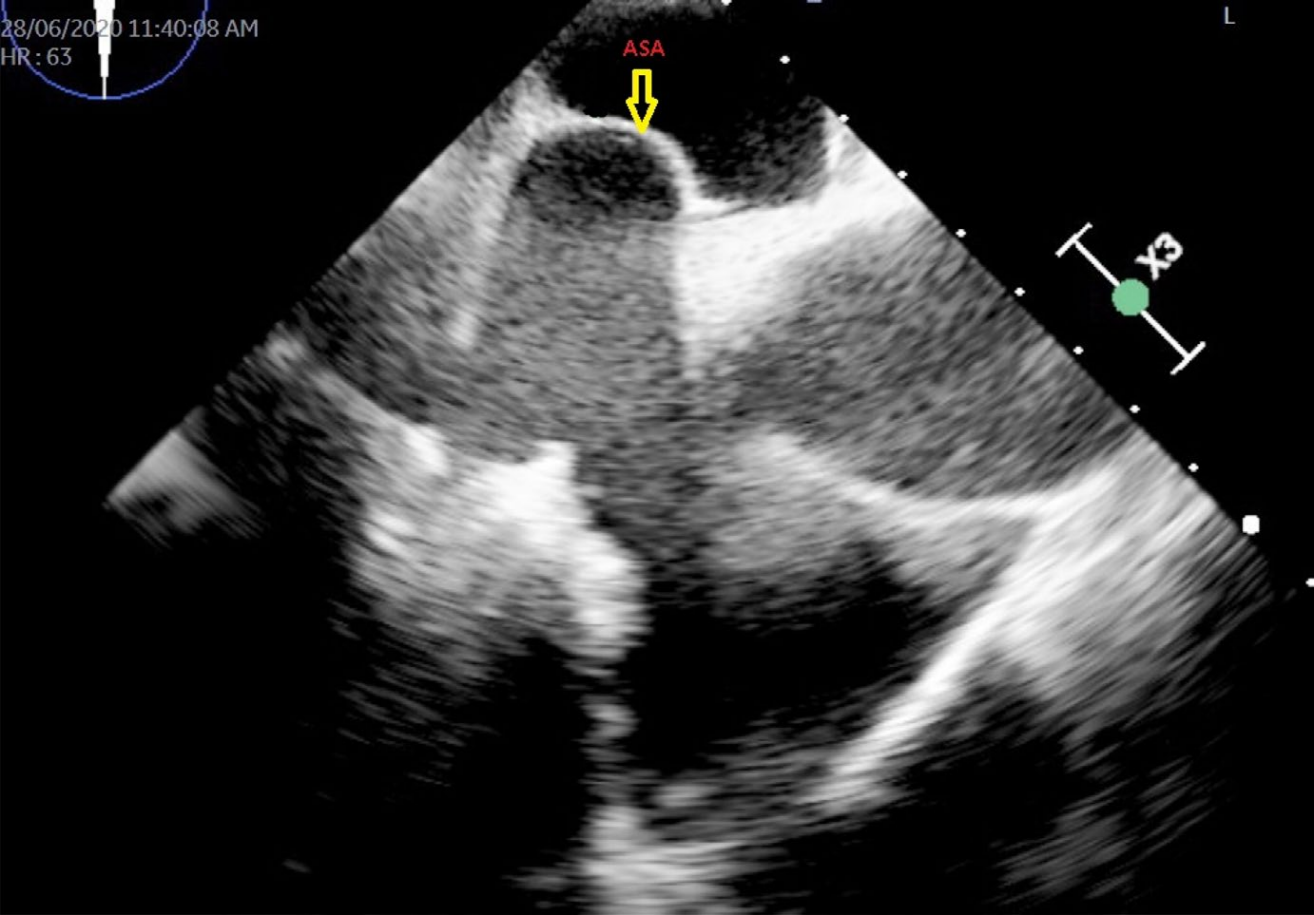
Transesophageal echocardiogram and yellow arrow showing atrial septal aneurysm (ASA)

**FIGURE 6:**
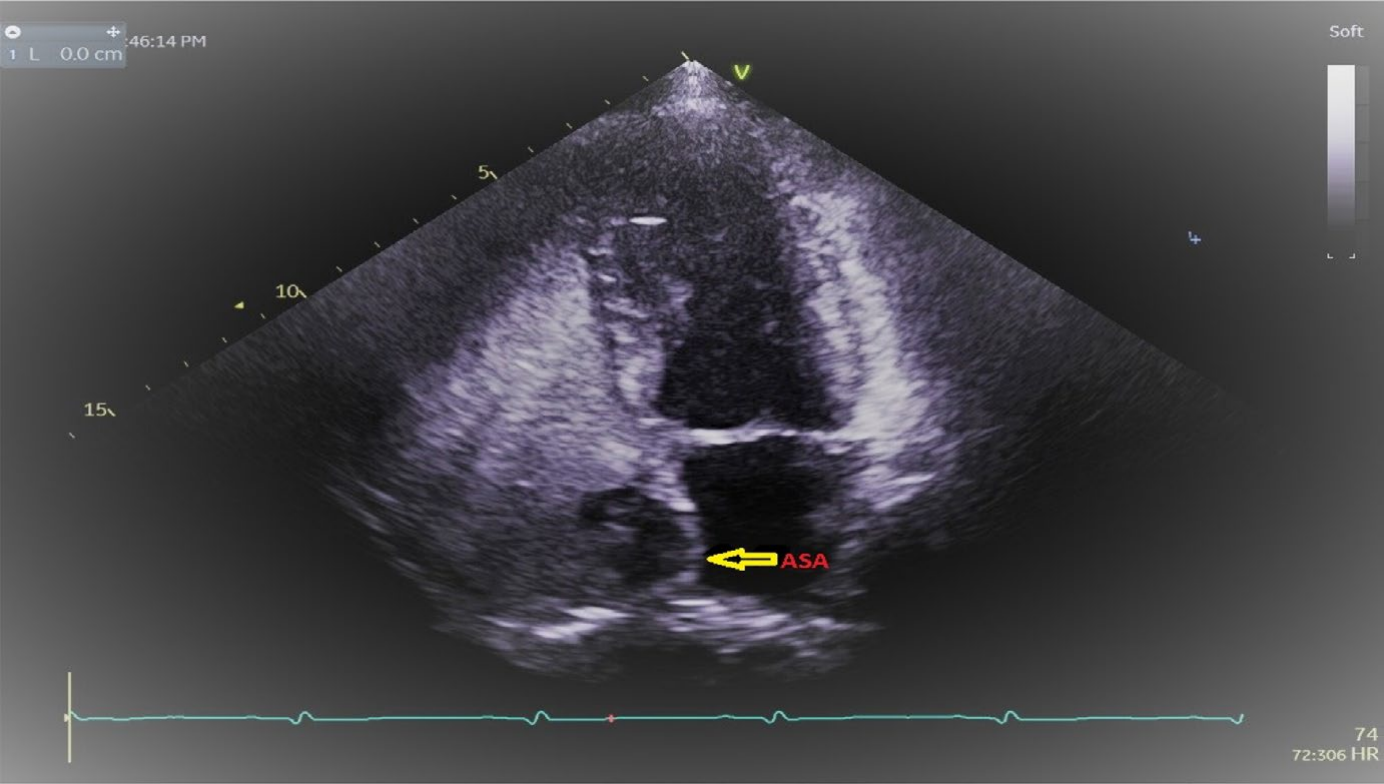
Transesophageal echocardiogram and yellow arrow showing atrial septal aneurysm (ASA)

Transesophageal echocardiography showed an atrial septal aneurysm but no patent foramen ovale or any other atrial septal defects (Figures 5 and 6). Bilateral lower limb Doppler imaging did not find deep vein thrombosis. A 48‐h Holter monitoring did not record atrial fibrillation. The patient was counseled about the conventional anticoagulation and direct oral anticoagulation (DOAC), the pros and cons of treatment were explained to him, and he chose DOAC subsequently. He was started on rivaroxaban 15 mg two times a day for 21 days, then 20 mg daily, and discharged on the same regimen. On 1‐week follow‐up from the date of discharge, no complications were noted, and he was compliant with his medication. Till writing this case, no complications or emergency visits for any reason were recorded.

## 3 DISCUSSION

Our patient suffered from ischemic colitis, which was confirmed by colonoscopy and tissue biopsy. His CT finding presented the embolic nature of the disease rather than diffused atherosclerosis, and the only risk factor was atrial septal aneurysm, which is a potential risk factor for cardiogenic embolism. After discussion with cardiology and gastroenterology specialists, our patient was diagnosed with arterial embolization with ischemic colitis secondary to an atrial septal aneurysm and was started on anticoagulation with rivaroxaban (off‐label).

Signs of atrial septal aneurysm can be either atrial arrhythmias or arterial embolisms. It can be a source of arrhythmic focus, leading to atrial tachycardias.^6^, ^7^ Arterial embolism is a well‐known complication, and different studies have shown a significant association between atrial septal aneurysm and arterial embolism.^1^, ^3^, ^4^ Echocardiography is the gold standard diagnostic tool for atrial septal aneurysms, which are usually discovered during routine tests or evaluation of cardioembolic stroke or arterial embolisms. Compared with transthoracic echocardiography, transesophageal echocardiography is considered more sensitive in detecting atrial septal aneurysm.^1^, ^6^ Other modalities such as cardiac tomography and cardiac magnetic resonance are also useful for diagnosis.^8^, ^9^ Treatment methods for this pathology vary according to the presentation and associated structural cardiac anomalies. In an asymptomatic patient with isolated atrial septal aneurysm, no specific treatment is required after ruling out an intracardiac thrombus. On the contrary, in a patient with cryptogenic stroke and an isolated atrial septal aneurysm, treatment options include medical therapy with antiplatelets or anticoagulation, as in the case of a recurrent stroke while taking antiplatelets. Rarely, surgical excision of the defect is considered in a patient with a recurrent stroke in whom antiplatelet or warfarin fails to prevent stroke recurrence or in patients with a large left‐to‐right shunt that leads to right heart enlargement. Percutaneous device closure is also rarely performed.^10^

Furthermore, in patients with atrial arrhythmias and embolic episodes, the preferred treatment is oral anticoagulation for secondary prevention. Most authors recommend traditional anticoagulation, such as in renal artery emboli limited to one of the renal arteries or segmental branches.^11^ Embolectomy is another option in the case of bilateral renal artery embolism, if the patient is suitable for the procedure. Intra‐abdominal thrombolysis is also a valid option in such cases.^12^ In a patient with stroke or transient ischemic attack, the American Heart Association/American Stroke Association has recommended using aspirin for secondary prevention, and warfarin can be used in high‐risk patients.^13^ In stroke associated with atrial septal aneurysm, anticoagulation was also employed.^3^, ^14^ Moreover, a paper reported a patient with paroxysmal atrial fibrillation who developed intracardiac thrombus and thereafter found to have an atrial septal aneurysm during a 3‐year follow‐up. The patient was started on warfarin. Six months later, no thrombus was found on repeated echocardiography, but the defect persisted despite adequate anticoagulation and surgery was then advised.^15^ To date, to our knowledge, no cases of ischemic colitis associated with an atrial septal aneurysm have been reported, and the use of DOACs in such cases and systemic arterial embolization secondary to atrial septal aneurysm has not been studied as well.

## 4 CONCLUSION

In a patient with ischemic colitis and no obvious risk factors, atrial septal aneurysm is a cardiac abnormality with thromboembolic potential and should be considered.

## CONFLICT OF INTEREST

The authors have no conflict of interest.

## AUTHOR CONTRIBUTIONS

Dr Eihab A. Subahi and Dr Mohamed A. Yassin wrote and edited the manuscript. Dr Narinder Kumar performed the echocardiography and provided us with labeled pictures of the cardiac lesion. Dr Abdulwahab M. Hamid performed the colonoscopy and provided us with labeled pictures of the colonoscopy. Dr Mohamed S. Elmahadi was in charge of clinical care.

## STATEMENT OF ETHICS

The patient consented to the publication of his case.

## Data Availability

The data that support the findings of this study are available from the corresponding author upon reasonable request.
